# Crystal structures of two hydrous sodium potassium molybdates: Na_3_K(MoO_4_)_2_(H_2_O)_9_ and NaK(MoO_4_)(H_2_O)

**DOI:** 10.1107/S2056989026004779

**Published:** 2026-05-12

**Authors:** Volker Kahlenberg

**Affiliations:** a University of Innsbruck, Institute of Mineralogy & Petrography, Innrain 52, A-6020 Innsbruck, Austria; Vienna University of Technology, Austria

**Keywords:** crystal structure, molybdate, hydrogen-bonding, mixed-alkali compound

## Abstract

Single-crystals of the hydrated sodium potassium orthomolybdate phases Na_3_K(MoO_4_)_2_(H_2_O)_9_, tris­odium potassium bis­(orthomolybdate) nona­hydrate, and NaK(MoO_4_)(H_2_O), sodium potassium orthomolybdate monohydrate, were obtained as by-products in a flux growth experiment aiming on the synthesis of silicates from the quaternary system Na_2_O–K_2_O–CaO–SiO_2_. The asymmetric unit of Na_3_K(MoO_4_)_2_(H_2_O)_9_ (space group *P*6_3_/*m*, *Z* = 2) comprises one Na^+^ atom and one water mol­ecule on a mirror plane, one K^+^ atom on a 6 axis, one Mo^6+^ cation and one O atom on a threefold rotation axis, and one O atom and one water mol­ecule in general sites. The crystal structure is built from isolated MoO_4_ tetra­hedra, which are linked by common corners to trimers containing three face-sharing Na(H_2_O)_4_O_2_ octa­hedra. Linkage between the heteropolyhedral groups is provided by potassium cations, which are coordinated by six water mol­ecules in form of trigonal prisms. The resulting layers are located at *z* = 1/4 and *z* = 3/4, respectively. Intra- and inter-layer hydrogen bonds between the water mol­ecules and the oxygen atoms of the MoO_4_ tetra­hedra consolidate the structure. The backbone of the crystal structure of NaK(MoO_4_)(H_2_O) (space group *P*2_1_2_1_2_1_, *Z* = 4) is made from chains of edge-sharing Na(H_2_O)_2_O_4_ octa­hedra running parallel [100], which are decorated by MoO_4_ tetra­hedra *via* corner sharing. Neighbouring heteropolyhedral chains are linked by additional potassium cations, which are coordinated by seven oxygen ligands and one water mol­ecule. Moreover, intra- and inter-chain hydrogen bonds exist.

## Abstract 2

The crystal structures of two hydrous mixed alkali orthomolybdates [Na_3_K(MoO_4_)_2_(H_2_O)_9_ and NaK(MoO_4_)(H_2_O)] are presented, including a discussion of structural similarities with related phases.

## 1. Chemical context

Oxidomolybdates offer the crystallographer a rich playground for structural investigations. This phenomenon can be attributed to the fact that the Mo atoms have the capacity to form covalent bonds with four, five or six oxygen atoms. The sharing of common oxygen atoms between the respective polyhedra facilitates the formation of larger negatively charged polyanions, including isolated groups, chains, and ultimately, three-dimensional frameworks (Krivovichev, 2009 ▸). Consequently, it is not unexpected that a multitude of molybdate structures have been documented in the present literature. If water mol­ecules are allowed to become incorporated, a further increase in the number of crystalline phases will be observed. In the event that the charge-compensating cations are restricted to group 1 elements, the current web version (5.5.0) of the Inorganic Crystal Structure Database (ICSD; Zagorac *et al.*, 2019 ▸) contains a total of 153 entries of anhydrous plus another 41 entries of hydrous phases. Hydrous alkali molybdates have also some industrial applications. For example, sodium molybdate dihydrate is used in corrosion science to protect metal surfaces, as it is a non-oxidizing anodic inhibitor (Vukasovich & Farr, 1986 ▸; Milošev, 2024 ▸). It is also used as a micronutrient to remedy problems in crops due to low molybdenum concentration in soils (O’Neil, 2013 ▸).

To the best of the author’s knowledge, only a few systematic investigations on the system Na_2_MoO_4_ – K_2_MoO_4_ – H_2_O have been performed so far. Mirzoev *et al.* (2007 ▸, 2010 ▸) studied the phase relations at 298 and 323 K and observed the following compounds: Na_2_(MoO_4_)(H_2_O)_2_, Na_3_K(MoO_4_)_2_, K_2_(MoO_4_) and Na_3_K(MoO_4_)_2_(H_2_O)_9_. It was not possible to obtain the nona­hydrate during the crystallization experiments conducted at 323 K. The latter compound was already mentioned in an earlier publication by Klevtsova *et al.* (1990 ▸), even though no detailed information on the synthesis conditions were given. Notably, no further hydrous mixed sodium potassium molybdate was found. As (i) the crystal structure of Na_3_K(MoO_4_)_2_(H_2_O)_9_ has not been reported or deposited in databases such as the ICSD, and (ii) the existence of a previously unknown phase with composition NaK(MoO_4_)(H_2_O) was observed in our experiments, a decision was taken to investigate both compounds using single-crystal X-ray diffraction in more detail.

## 2. Structural commentary

### 2.1. Na_3_K(MoO_4_)_2_(H_2_O)_9_

**Figure 1:**
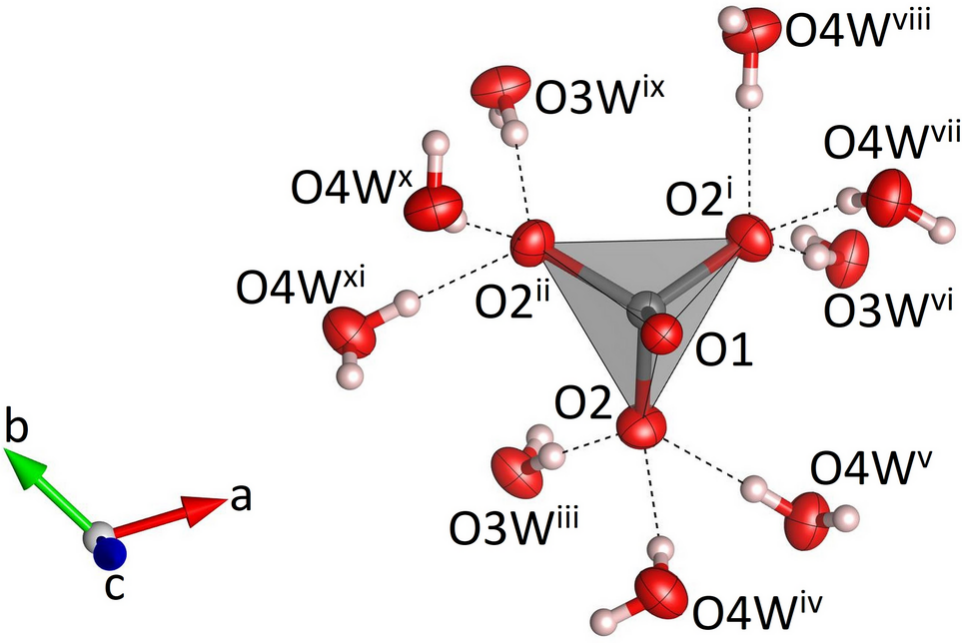
Single MoO_4_ tetra­hedron in Na_3_K(MoO_4_)_2_(H_2_O)_9_ and the hydrogen bonds (dashed black lines) involving the corresponding oxygen atoms. Molybdenum, oxygen and hydrogen atoms are shown in gray, red and white, respectively. Displacement ellipsoids are given at the 70% probability level except for H atoms, which are shown with an arbitrary radius. [Symmetry codes: (i) 1 − *y*, *x* − *y* + 1, *z*; (ii) −*x* + *y*, 1 − *x*, *z*; (iii) *x* − *y*, *x*, 1 − *z*; (iv) *x*, *y*, 1 + *z*; (v) *y*, −*x* + *y*, 1 − *z*; (vi) 1 − *x*, 1 − *y*, 1 − *z*; (vii) 1 − *y*, *x* − *y* + 1, 1 + *z*; (viii) *x* − *y* + 1, 1 + *x*, 1 − *z*; (ix) *y*, 1 − *x* + *y*, 1 − *z*; (*x*) −*x* + *y*, 1 − *x*, 1 + *z*; (xi) −*x*, 1 − *y*, 1 − *z*.]

**Figure 2:**
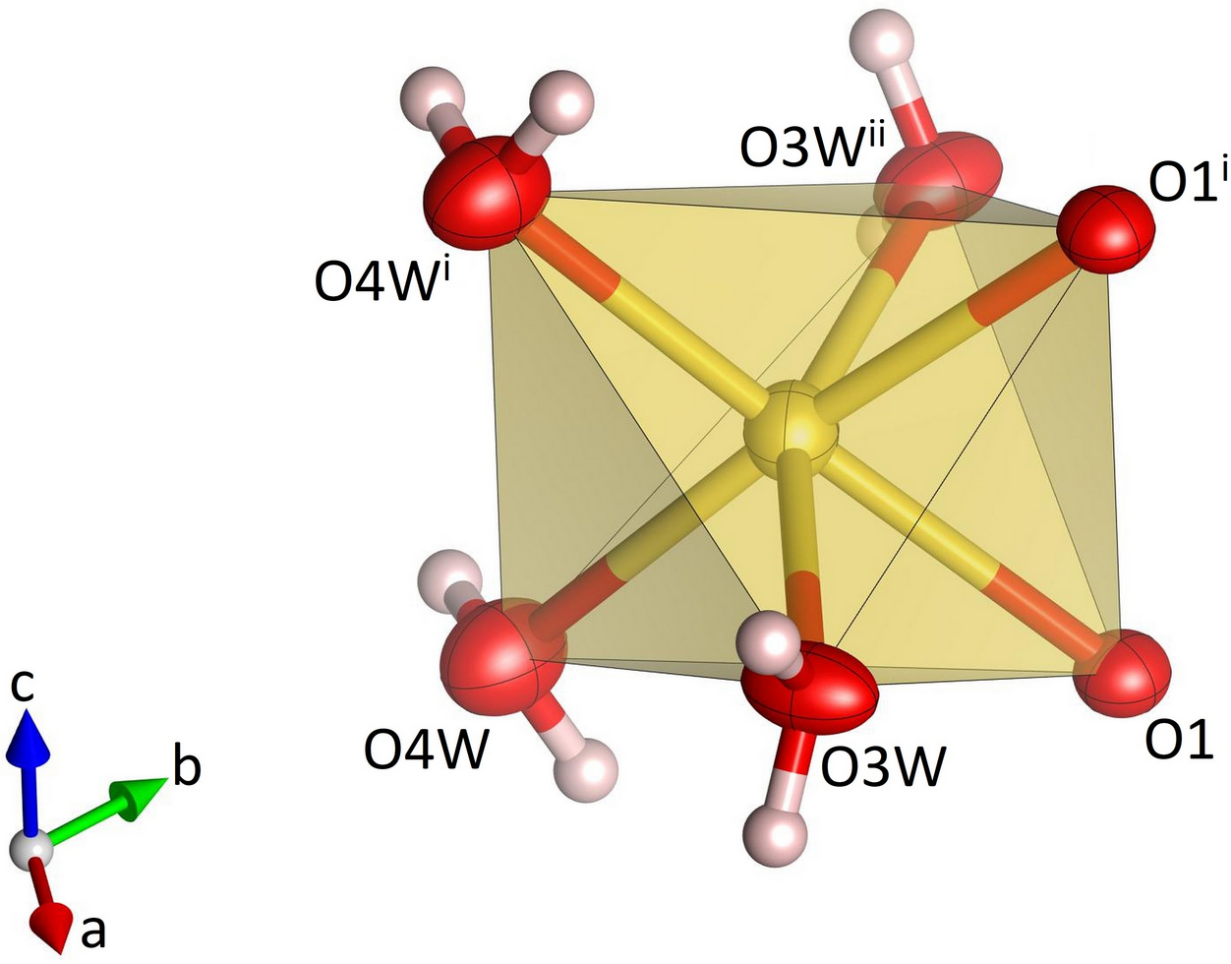
Single Na(H_2_O)_4_O_2_ octa­hedron in Na_3_K(MoO_4_)_2_(H_2_O)_9_. Sodium, oxygen and hydrogen atoms are shown in yellow, red and white, respectively. Displacement ellipsoids are given at the 70% probability level except for H atoms, which are shown with an arbitrary radius. [Symmetry codes: (i) *x*, *y*, −*z* + 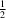; (ii) −*x* + *y*, 1 − *x*, *z*.]

**Figure 3:**
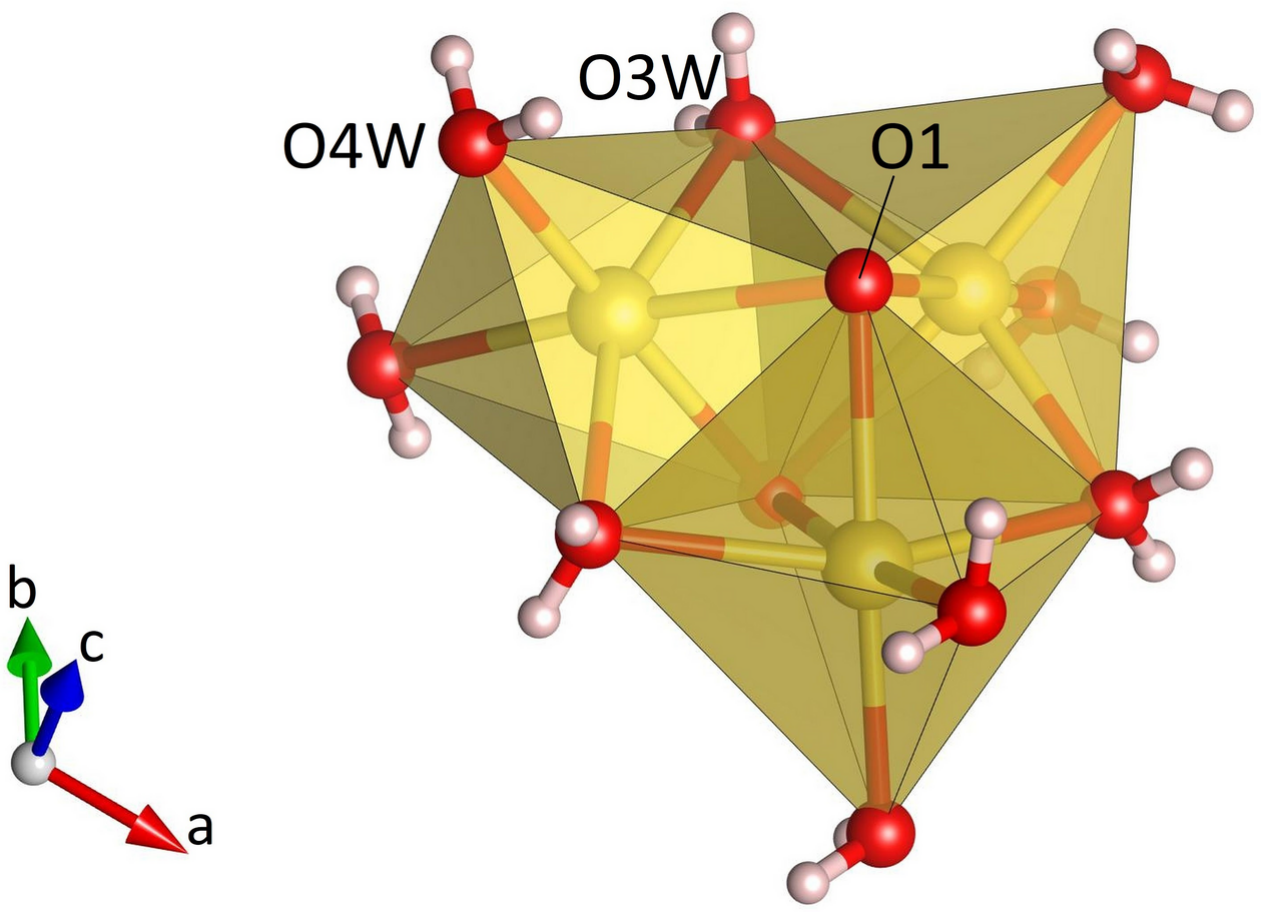
Side view of a single Na_3_O_2_(H_2_O)_9_ group in Na_3_K(MoO_4_)_2_(H_2_O)_9_ formed by the condensation of three face-sharing octa­hedra.

**Figure 4:**
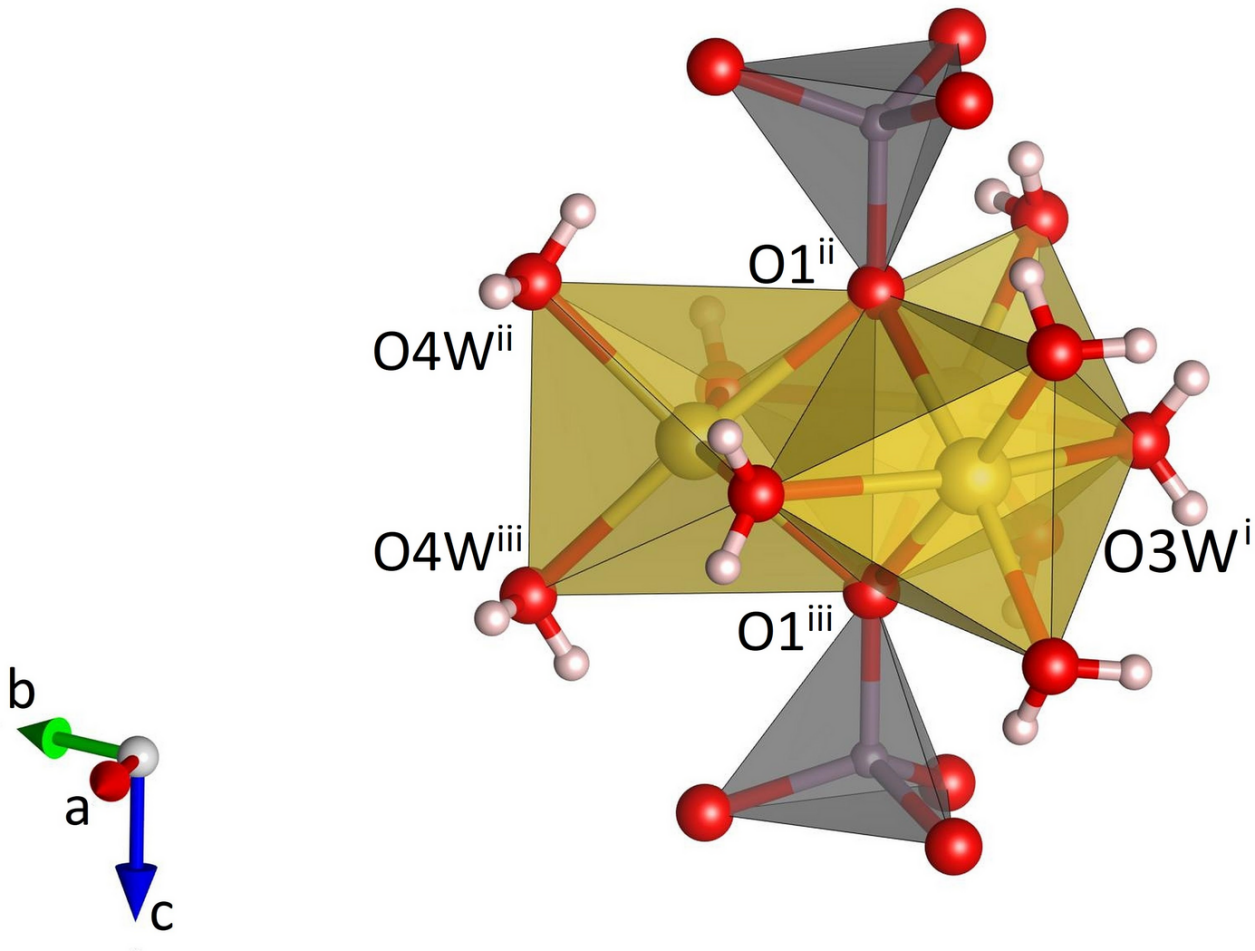
Side view of a single decorated Na_3_(H_2_O)_9_(MoO_4_)_2_ group in Na_3_K(MoO_4_)_2_(H_2_O)_9_ formed by the linkage to two MoO_4_ tetra­hedra on both sides. [Symmetry codes: (i) *y*, −*x* + *y*, 1 − *z*; (ii) 1 − *x*, 1 − *y*, *z* + 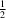; (iii) 1 − *x*, 1 − *y*, 1 − *z*.]

**Figure 5:**
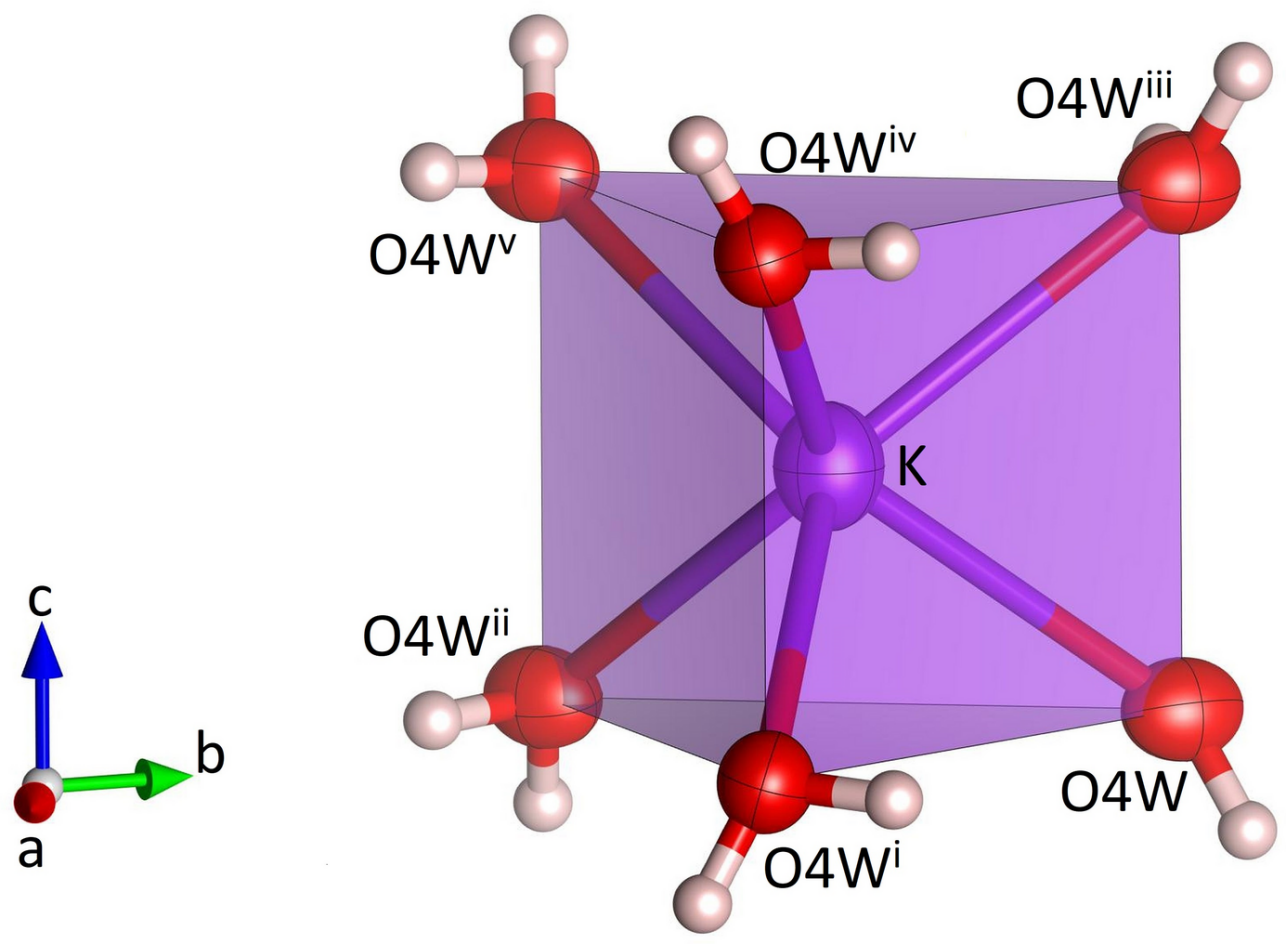
Single K(H_2_O)_6_ prism in Na_3_K(MoO_4_)_2_(H_2_O)_9_. Potassium, oxygen and hydrogen atoms are shown in purple, red and white, respectively. Displacement ellipsoids are given at the 70% probability level except for H atoms, which are shown with an arbitrary radius. [Symmetry codes: (i) −*x* + *y*, −*x*, *z*; (ii) −*y*, *x* − *y*, *z*; (iii) −*x* + *y*, −*x*, −*z* + 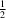; (iv) *x*, *y*, −*z* + 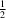; (v) −*y*, *x* − *y*, −*z* + 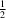.]

**Figure 6:**
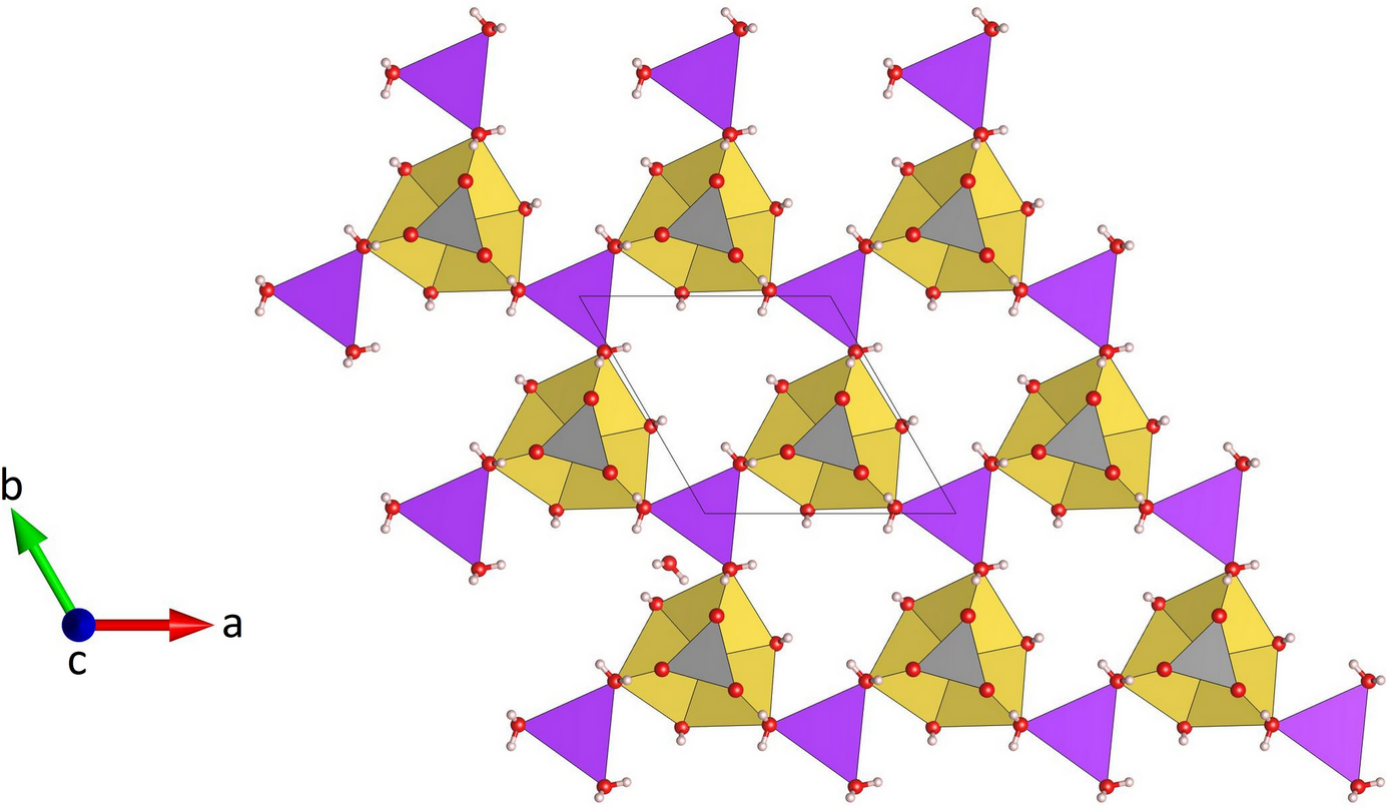
Projection of a single heretopolyhedral layer at *z* = 3/4 in Na_3_K(MoO_4_)_2_(H_2_O)_9_ parallel to [001].

**Figure 7:**
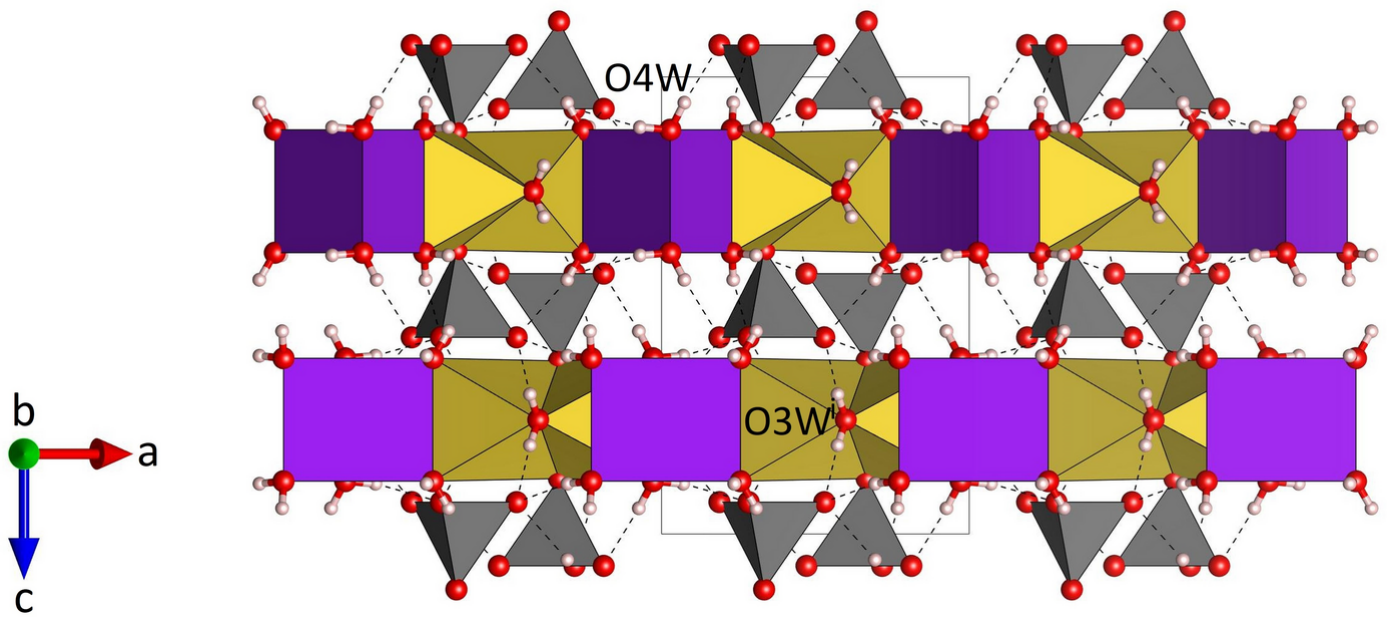
Projection of the whole crystal structure of Na_3_K(MoO_4_)_2_(H_2_O)_9_ parallel to [010]. Intra- and inter-layer hydrogen bonds are shown with black dashed lines. [Symmetry code: (i) 1 − *x*, −*y*, 1 − *z*.]

Na_3_K(MoO_4_)_2_(H_2_O)_9_ is isostructural with Na_3_Rb(MoO_4_)_2_(H_2_O)_9_ (ICSD-entry no. 39293, based on the data published by Klevtsova *et al.*, 1990 ▸) and Na_3_K(WO_4_)_2_(H_2_O)_9_ (ICSD-entry no. 39294; Klevtsova *et al.*, 1990 ▸). The compound crystallizes in the hexa­gonal space group *P*6_3_/*m*. The unit cell contains two formula units. The structure comprises insular MoO_4_ tetra­hedra (Fig. 1 ▸) occupying the Wyckoff-position 4*f* (site symmetry 3..). The Mo—O bond lengths range from 1.753 (3) to 1.768 (2) Å (Table 1 ▸). The distances between the Mo atoms and the three basal O2 atoms are slightly larger than the corresponding bond lengths to the apical O1 atom. The average value <Mo—O> = 1.764 Å is in perfect agreement with the value reported by Gagné & Hawthorne (2020 ▸) for hexa­valent Mo^[4]^ obtained from a bond-length dispersion analysis of more than 1700 individual bonds. The six O—Mo—O angles have values that are very close to the ideal tetra­hedral angle of 109.5°. The degree of tetra­hedral distortion can be qu­anti­fied using the following two parameters: quadratic elongation (QE) and angle variance (AV) (Robinson *et al.*, 1971 ▸). The numerical values for these parameters reflect the very low degree of distortion: QE = 1.000 and AV = 0.14. The sodium cations are located on mirror planes perpendicular to [001] (Wyckoff-position 6*h*) and are octa­hedrally coordinated by four water mol­ecules and two oxygen atoms belonging to two symmetry-equivalent MoO_4_ units (Fig. 2 ▸). Three adjacent octa­hedra form a Na_3_O_2_(H_2_O)_9_ group, in which two faces of each octa­hedron are shared by the other two octa­hedra belonging to the same trimer (Fig. 3 ▸). The faces are defined by two O1 atoms and one O3*W* mol­ecule. Notably, the O1–O1 edge is a common element of all three faces. The barycentres of the group (site symmetry 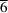), located at the midpoint of the central O1–O1 edge, have the fractional coordinates of 1/3 2/3 1/4 and 2/3 1/3 3/4, respectively. As may be anti­cipated, the bonds between Na and the two terminal (unshared) O4*W* oxygen atoms of the Na(H_2_O)_4_O_2_ octa­hedra are significantly shorter [2.343 (2) Å] than the corresponding bond lengths to the bridging O atoms of the group (average value = 2.416 Å). The corresponding distortion parameters have values of QE = 1.018 and AV = 64.18. The trimers are decorated by MoO_4_ tetra­hedra on both sides sharing a common oxygen atom O1, which implies that the tetra­hedra point in opposite directions (Fig. 4 ▸). The resulting heteropolyhedral unit has the composition Na_3_(H_2_O)_9_(MoO_4_)_2_. The potassium cations (Wyckoff-position 2*a*) are coordinated by six water mol­ecules (Fig. 5 ▸). The coordination polyhedron corresponds to a trigonal prism with site symmetry 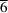. A single K(H_2_O)_6_ prism shares three O4*W*–O4*W* edges with Na_3_(H_2_O)_9_(MoO_4_)_2_ groups that are directly adjacent. Consequently, mixed-polyhedral layers are formed at *z* = 1/4 and *z* = 3/4 that are parallel to (001) (Fig. 6 ▸). Further linkage between the polyhedra is facilitated by hydrogen bonding. A projection of the whole structure parallel to [010] is shown in Fig. 7 ▸. Indeed, each of the three basal oxygen atoms (O2) of the tetra­hedra are acceptors of one inter-layer (O2⋯H42) and two intra-layer (O2⋯H41, O2⋯H3) hydrogen bonds (Table 2 ▸, Figs. 1 ▸ and 7 ▸). The two symmetry-equivalent hydrogen atoms associated with O3 connect the corresponding Na(H_2_O)_4_O_2_ octa­hedra with two directly adjacent heteropolyhedral layers (Fig. 7 ▸). The range of distances between the relevant donors and acceptors is from 2.746 (2) to 2.913 (3) Å. Therefore, all hydrogen bonds can be classified as of medium strength (Steiner, 2002 ▸).

### 2.2. NaK(MoO_4_)(H_2_O)

**Figure 8:**
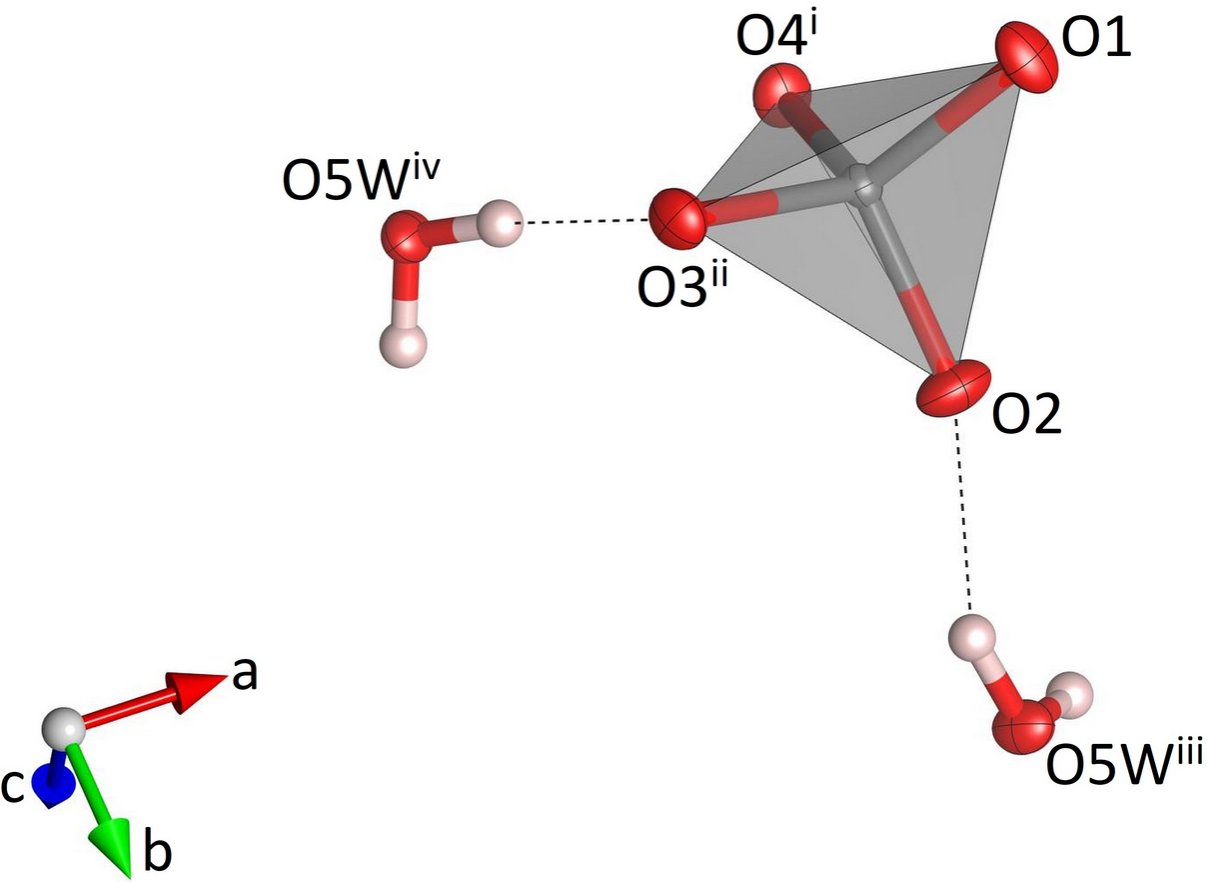
Single MoO_4_ tetra­hedron in NaK(MoO_4_)(H_2_O) and the hydrogen bonds (dashed black lines) involving the corresponding oxygen atoms. Molybdenum, oxygen and hydrogen atoms are shown in gray, red and white, respectively. Displacement ellipsoids are given at the 70% probability level except for H atoms, which are shown with an arbitrary radius. [Symmetry codes: (i) *x*, 1 − *y*, *z*; (ii) *x* − 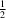, −*y* + 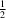, 1 − *z*; (iii) 1 − *x*, *y * + 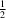, −*z* + 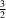; (iv) −*x* + 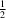, −*y*, *z* − 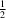
*.*]

**Figure 9:**
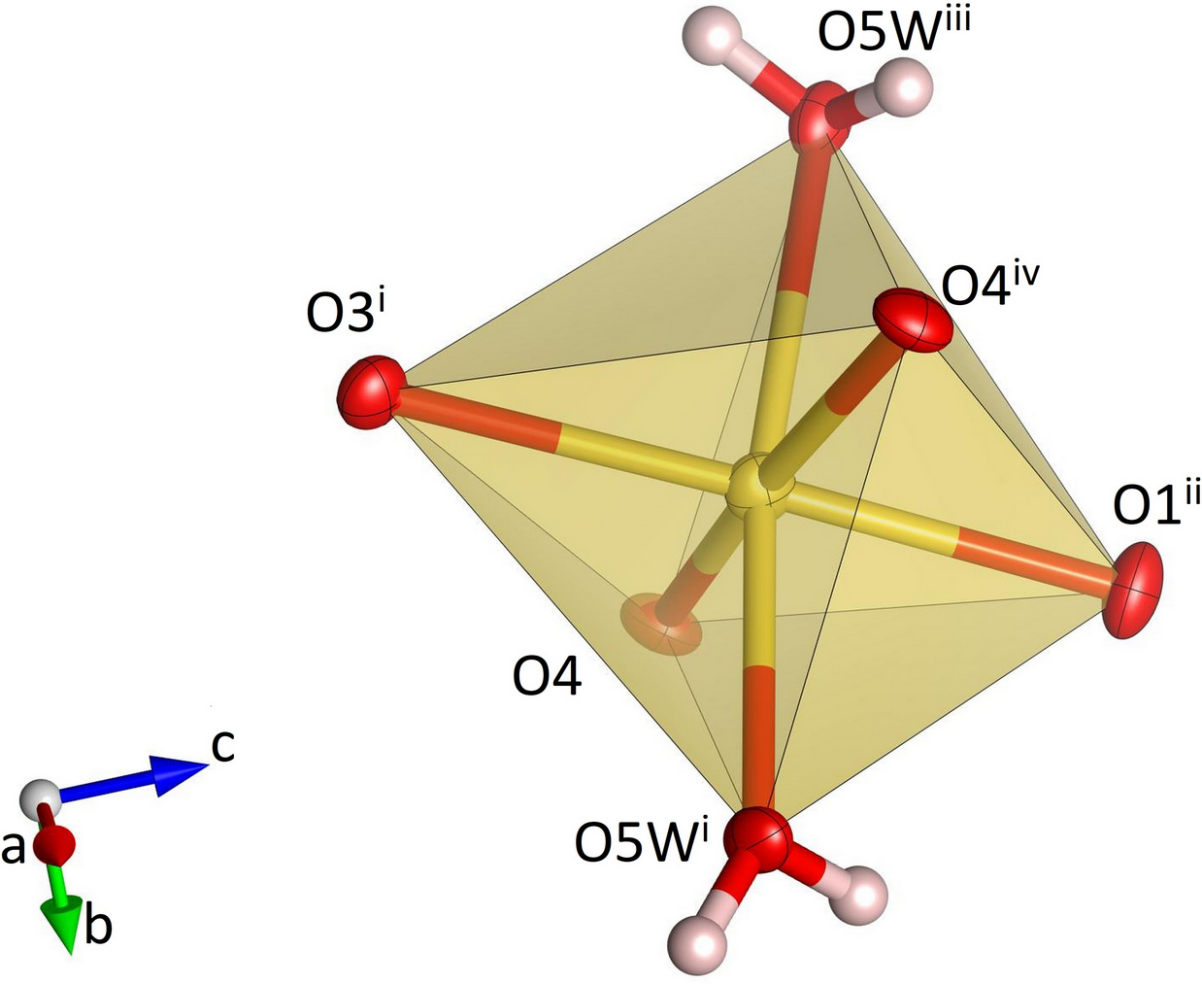
Single Na(H_2_O)_2_O_4_ octa­hedron in NaK(MoO_4_)(H_2_O). Sodium, oxygen and hydrogen atoms are shown in yellow, red and white, respectively. Displacement ellipsoids are given at the 70% probability level except for H atoms, which are shown with an arbitrary radius. [Symmetry codes: (i) −*x* + 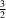, 1 − *y*, *z* − 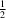; (ii) −*x* + 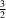, 1 − *y*, *z* + 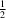; (iii) 1 − *x*, *y* + 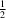, −*z* − 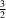; (iv) *x* + 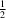, −*y* + 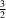, 1 − *z*.]

**Figure 10:**
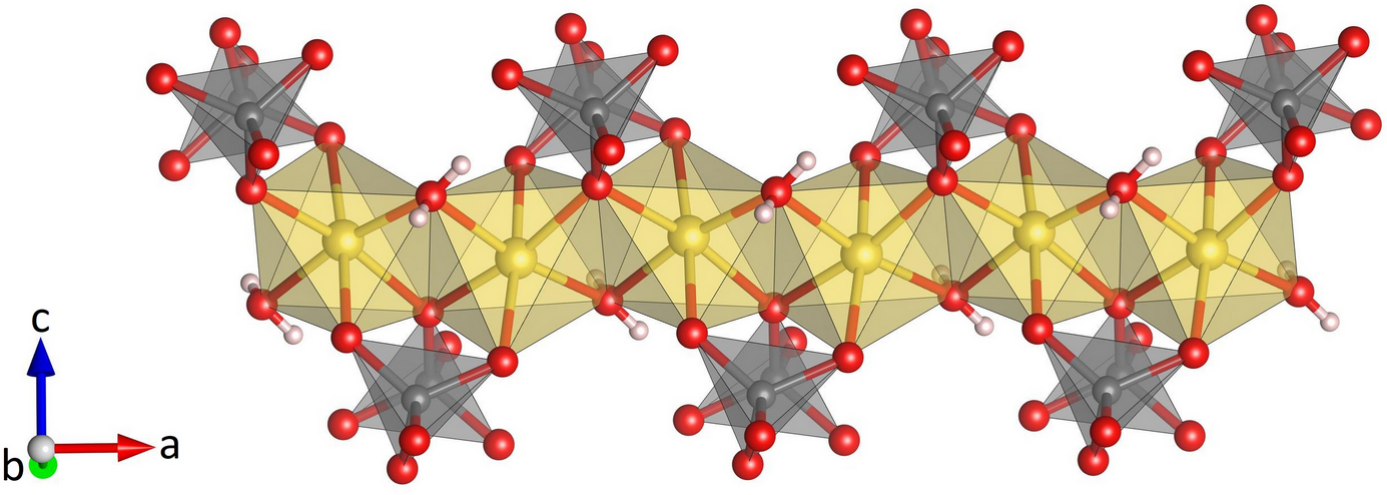
Side view of a heteropolyhedral Na(MoO_4_)_2_(H_2_O) chain in NaK(MoO_4_)(H_2_O).

**Figure 11:**
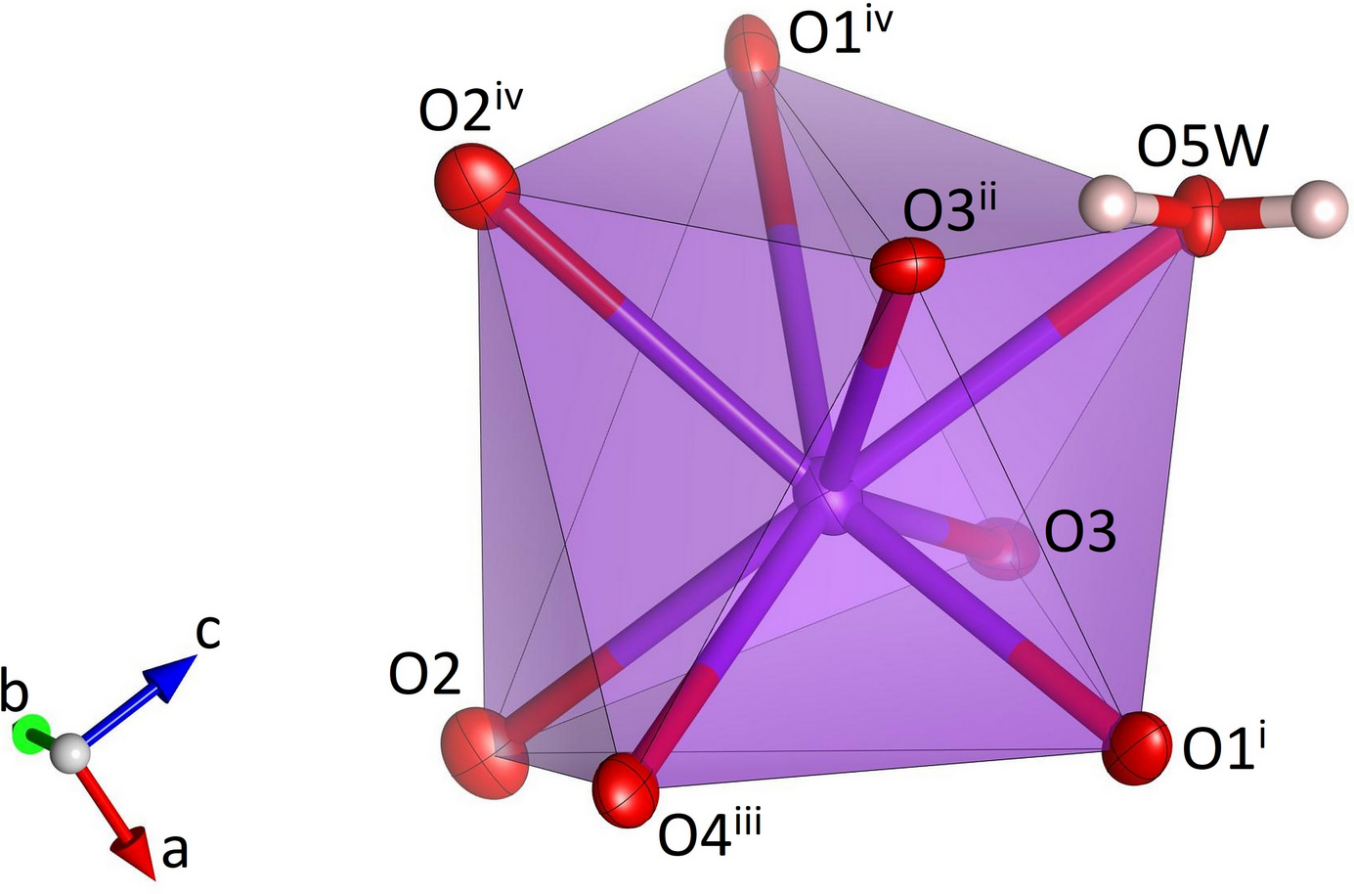
Single coordination polyhedron for potassium in NaK(MoO_4_)(H_2_O). Potassium, oxygen and hydrogen atoms are shown in purple, red and white, respectively. [Symmetry codes: (i) −*x* + 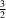, −*y*, *z* + 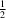; (ii) 1 − *x*, *y* − 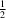, −*z* + 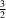; (iii) *x*, *y* − 1, *z*; (iv) *x* − 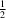, −*y* + 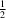, 1 − *z*.]

**Figure 12:**
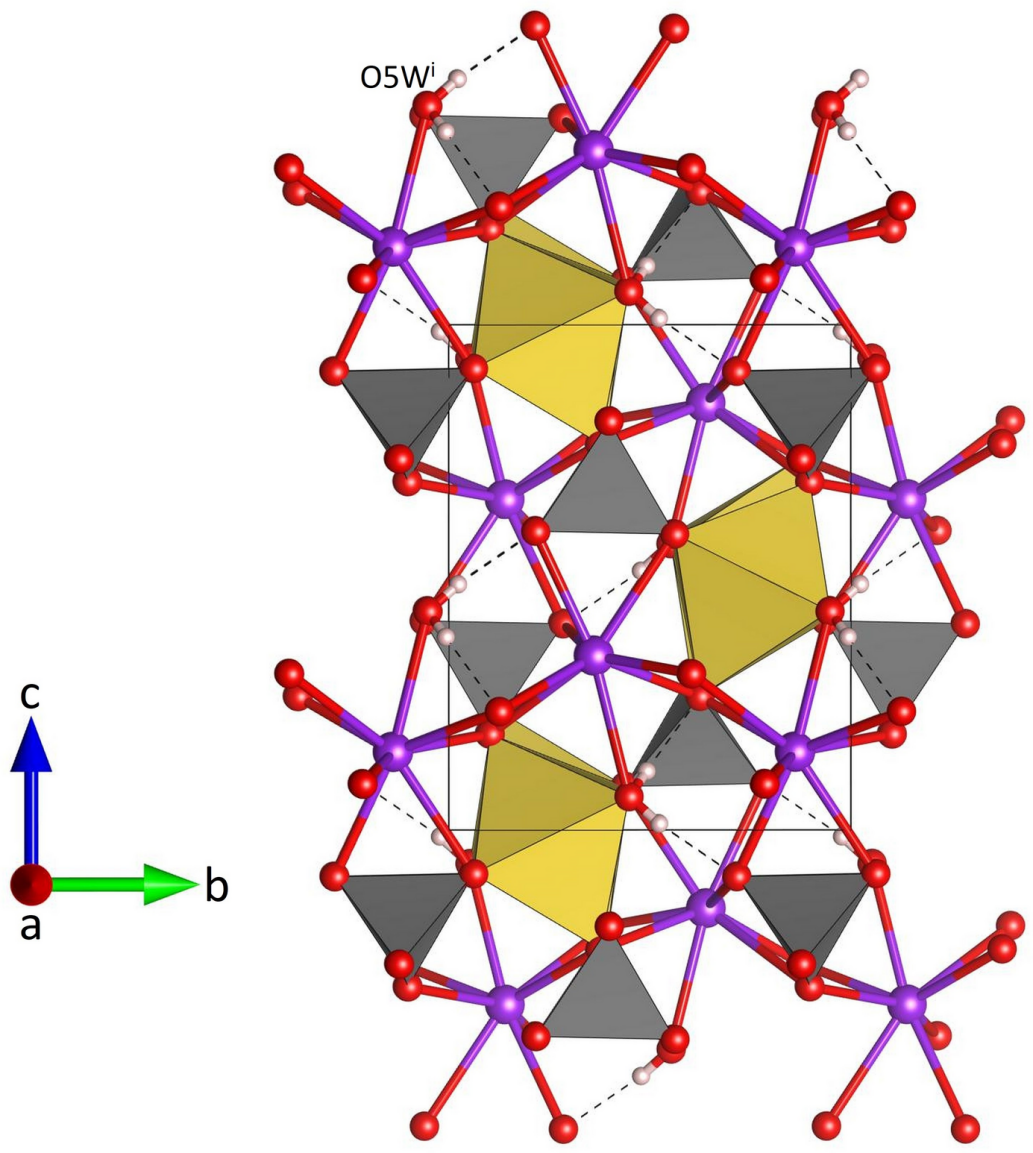
Projection of the whole crystal structure of NaK(MoO_4_)(H_2_O) parallel to [100]. Intra- and inter-chain hydrogen bonds are shown with black dashed lines. [Symmetry code: (i) -*x* + 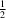, −*y*, *z* + 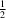.]

NaK(MoO_4_)(H_2_O) crystallizes in the non-centrosymmetric ortho­rhom­bic space group *P*2_1_2_1_2_1_ and comprises four formula units in the unit cell. The MoO_4_ tetra­hedron (Fig. 8 ▸) shows Mo—O bond lengths between 1.754 (3) and 1.785 (3) Å (Table 3 ▸) with distortion parameters of QE = 1.0024 and AV = 9.0515. The spread and average value of the Mo—O bonds are consistent with the literature data (Gagné & Hawthorne, 2020 ▸). The sodium cations are coordinated by six ligands involving two water mol­ecules and four oxygen atoms (Fig. 9 ▸). The water mol­ecules are located in a *trans* position. Bond lengths within the Na(H_2_O)_2_O_4_ unit range from 2.342 (3) to 2.453 (3) Å with distortion parameters of QE = 1.0129 and AV = 42.43. Adjacent octa­hedra share two *trans* edges to form chains running parallel [100] (Fig. 10 ▸). The corners of neighbouring octa­hedra (O1, O3) are linked via MoO_4_ groups that assume a staggered configuration along the chain direction. The comparatively short O1–O3 edge of the attached rigid tetra­hedra acts as a clamp that induces a cooperative rotation/distortion of the octa­hedra. Additional MoO_4_ tetra­hedra are connected to one of the vertices of the edge that is common to adjacent octa­hedra inside the chain. Again, the units adopt a staggered configuration when viewed along [100]. The resulting chemical composition of the heteropolyhedral chains corresponds to Na(MoO_4_)_2_(H_2_O). Charge compensation is provided by potassium cations, which are incorporated in the voids between the chains. In more detail, each K^+^ ion is coordinated by eight next oxygen ligands including one water mol­ecule (Fig. 11 ▸). Up to 3.2 Å, the K—O bond lengths vary between 2.724 (3) and 3.198 (3) Å (average value: 2.897 Å). The <K—O> distance is in excellent agreement with the value of 2.894 Å reported by Gagné & Hawthorne (2016 ▸) for K^[8]^. The hydrogen atoms of the water mol­ecule O5*W* form single hydrogen bonds with the oxygen atoms O2 and O3, respectively (Fig. 8 ▸, Table 4 ▸). The O3⋯H52 inter­action represents an intra-chain bond, whilst the corresponding O2⋯H51 hydrogen bond connects neighbouring heteropolyhedral chains. The donor–acceptor distances are indicative of hydrogen bonds of medium strength (Steiner, 2002 ▸). A projection of the whole crystal structure of NaK(MoO_4_)(H_2_O) is presented in Fig. 12 ▸.

### 2.3. Bond-valence sums

For both alkali molybdate hydrates, calculations of bond-valence sums (BVS) in valence units (v.u.) were performed using the parameter sets for Mo—O, Na—O and K—O listed by Brown & Altermatt (1985 ▸) to verify the correctness of the structure models. The BVS values for all atomic sites are summarized in Tables 5 ▸ and 6 ▸. The calculations were performed both with and without the contributions of the hydrogen atoms of the water mol­ecules. Following the suggestion of Hawthorne (1997 ▸), the effect of the hydrogen atoms has been taken into consideration by attributing 0.8 v.u. to the donor oxygen atom and 0.2 v.u. to the acceptor oxygen of the hydrogen bond. The results generally compare well with the expected values of 1.00 v.u. for K, 6.00 for Mo and 2.00 v.u. for O. However, it is noteworthy, that the sodium cations show slightly larger deviations from 1.00 v.u. (BVS values of 1.22 v.u.), indicating an overbonding; that is, the octa­hedral voids occupied by this type of alkali cation are slightly too small.

### 2.4. Thermal expansion

**Figure 13:**
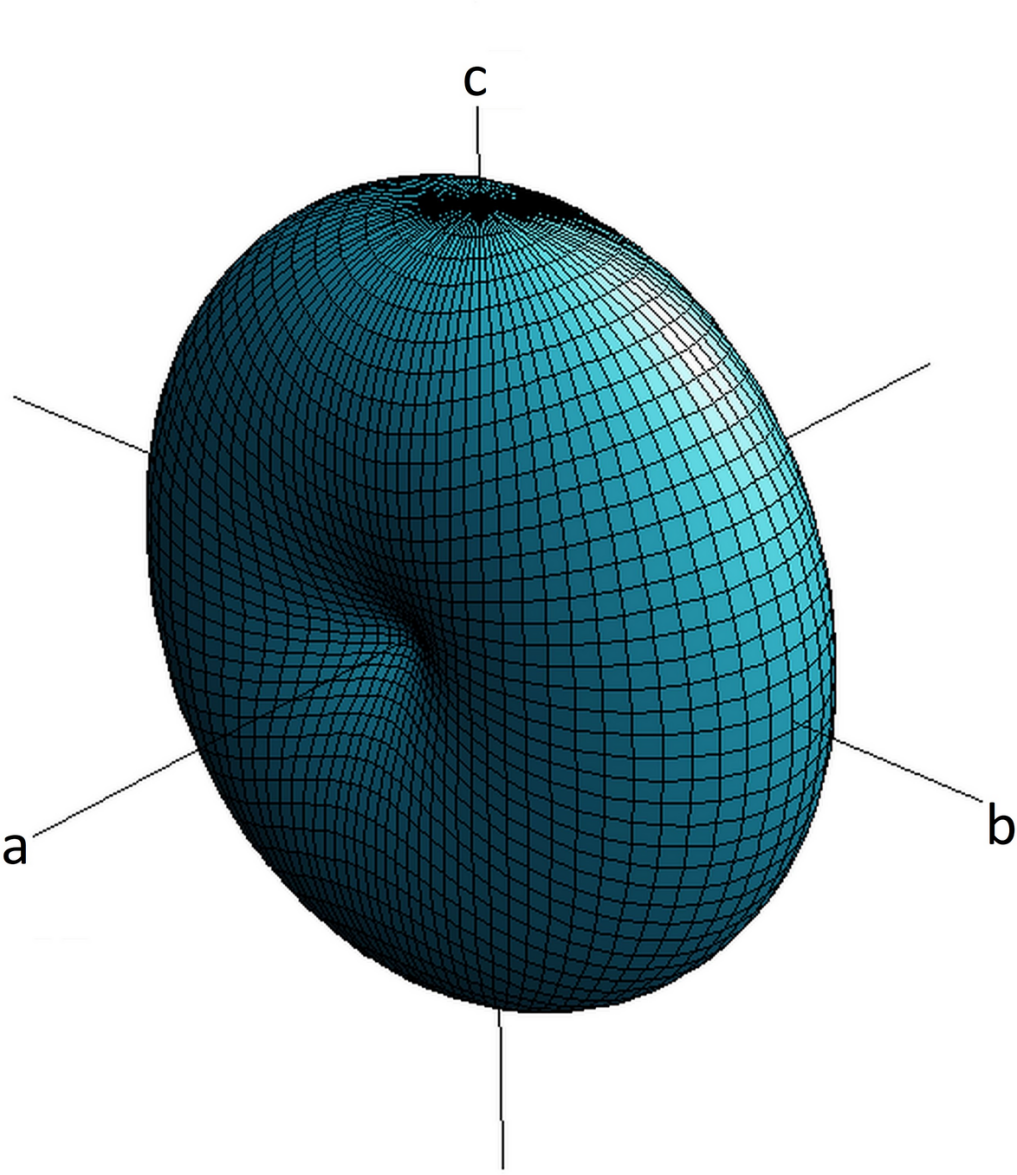
Three-dimensional representation surface of the average thermal expansion tensor of NaK(MoO_4_)(H_2_O) in the inter­val between 193 and 296 K.

Unfortunately, the crystals of Na_3_K(MoO_4_)_2_(H_2_O)_9_ were not stable at ambient conditions for more than a couple of hours. Therefore, it was decided to determine the thermal expansion tensor only for NaK(MoO_4_)(H_2_O). For the data collection at 193 K, the crystal was mounted on a LithoLoop (Mol­ecular Dimensions) using a drop of Paratone-N oil (Hampton Research) and immersed in a cold air stream generated by an Oxford Cryosystems Desktop Cooler. The very same sample was then affixed to the tip of a glass fibre with fingernail hardener, in order to obtain data at 296 K. As the refined structural parameters of the room-temperature investigation are essentially identical to those of the low-temperature study, they will not be reported in detail. Instead, the focus will be on determining the thermal expansion tensor from the two sets of lattice parameters. Please refer to Table 7 ▸ for the respective values for 193 K. The corresponding values at ambient temperature are as follows: *a* = 6.4859 (7) Å, *b* = 8.1025 (8) Å, and *c* = 10.1835 (12) Å. The average thermal expansion tensor α_ij_ for a given temperature inter­val, Δ*T*, can be calculated from the thermal strain tensor ɛ_ij_ and the relationship α_ij =_ ɛ_ij_/Δ*T*. Due to the ortho­rhom­bic symmetry restrictions, the off-diagonal terms of the symmetric second-rank tensor α_*ij*_ with *i* ≠ *j* must be strictly zero. The remaining three components can be obtained from the following expressions: ɛ_11_ = (*a*/*a*_0_) −1, ɛ_22_ = (*b*/*b*_0_) −1 and ɛ_33_ = (*c*/*c*_0_) −1. Notably, the lattice parameters with the suffix ‘zero’ pertain to the low-tem­per­ature data. In consequence, the off-diagonal components have the following values: α_11_ = 11 (1) × 10^−6^, α_22_ = 39 (1) × 10^−6^, and α_33 =_ 42 (1) × 10^−6^. From the comparison of the numerical values it is obvious that the thermal expansion shows a pronounced anisotropy. The expansion along [100], that is, along the rigid chain-like building blocks of the crystal structure, is about a factor four smaller than along [010] and [001], respectively. Notably, α_22_ and α_33_ are equal within two standard deviations. By plotting the values of the thermal expansion tensor as a function of all directions one obtains a convenient geometric representation of the anisotropic behaviour of the tensor in the form of a surface in three-dimensional space (Fig. 13 ▸).

## 3. Database survey

As mentioned above, Na_3_K(MoO_4_)_2_(H_2_O)_9_ is isotypic with the corresponding rubidium compound (Klevtsova *et al.*, 1990 ▸). For the calculation of several qu­anti­tative descriptors for the characterization of the degree of similarity, the program *COMPSTRU* (de la Flor *et al.*, 2016 ▸) was employed. After a transformation according to **a′** = –**b**, **b′** = –**a**, and **c′** = –**c** the structure of Na_3_Rb(MoO_4_)_2_(H_2_O)_9_ was transformed to the most similar configuration of Na_3_K(MoO_4_)_2_(H_2_O)_9_. The calculations revealed the following displacements (in Å) between the corresponding atom pairs in both phases: Mo: 0.011; Na: 0.034; K: 0.000; O1: 0.011; O2: 0.040; O3: 0.063; O4: 0.124. The measure of similarity (Δ) as defined by Bergerhoff *et al.* (1999 ▸) has a value of 0.030. Notably, the most pronounced shifts occur between the oxygen atoms of the water mol­ecules surrounding the potassium cations. The degree of lattice distortion *S* is related to the spontaneous strain that can be obtained from a comparison of the unit-cell parameters of both phases. In more detail, it is the square root of the sum of the squared eigenvalues of the strain tensor divided by 3. For the given two structure descriptions, *S* has a value of 0.0075. To the best of the author’s knowledge, the presence of the Na_3_O_2_(H_2_O)_9_ or more generally, M_3_φ_11_ units built on three octa­hedra sharing two faces, is rather an exception. According to Pauling’s third rule (Pauling, 1929 ▸), shared faces between coordination polyhedra dramatically decreases the stability of a crystal structure. It is noteworthy, that one of the cesium suboxides contains equivalent anion-centred units with composition O_3_Cs_11_ (Simon *et al.*, 1978 ▸).

NaK(MoO_4_)(H_2_O) represents a new structure type. Nevertheless, its characteristic ^[6]^M(TO_4_)_2_φ chains (φ: H_2_O, OH, F) have been already observed in a number of phosphate-, arsenate- and vanadate-based minerals including wherryite [Pb_7_Cu_2_(SO_4_)_4_(SiO_4_)_2_(OH)_2_; Cooper & Haw­thorne, 1994 ▸] and brackebuschite [Pb_2_(Mn^3+^,Fe^3+^)(VO_4_)_2_(OH); Foley *et al.*, 1997 ▸], for example. Further representatives can be found in the review publications of Hawthorne (1998 ▸) and Lussier & Hawthorne (2021 ▸) on decorated and undecorated chains of edge-sharing octa­hedra. The present phase is the first pure molybdate member of this group of compounds. Further examples of low-hydrated mixed alkali hydrates containing sodium include NaLi(MoO_4_)(H_2_O)_2_ (Makitova *et al.*, 1990 ▸) and NaCs(MoO_4_)(H_2_O)_2_ (Klevtsov *et al.*, 1997 ▸). However, these two materials are structurally not related to the present compound. The first phase is composed of units of two Na(H_2_O)_2_O_4_ octa­hedra and two Li(H_2_O)_2_O_3_ tetra­gonal pyramids sharing common edges, which are linked by MoO_4_ tetra­hedra. The structural backbones of the latter compound comprise chains of face-sharing Na(H_2_O)_4_O_2_ octa­hedra, which are decorated with MoO_4_ tetra­hedra. Only very recently, chemically related Na_2_(MoO_4_)(H_2_O)_2_, a synthetic compound with some relevance in industrial inorganic chemistry, has also been found in nature in a fumarole deposit of the Tolbachik volcano, Kamchatka, Russia. The new mineral was named natromolybdite (Pekov *et al.*, 2025 ▸). It is possible, that a natural equivalent of synthetic NaK(MoO_4_)(H_2_O) can be found in similar petrographic environments. According to the present investigation, NaK(MoO_4_)(H_2_O) exhibits high solubility in water and crystallizes readily. This may provide an opportunity for the targeted growth of larger crystals of this acentric phase, which could be further studied for potential applications in nonlinear optics, for example.

## 4. Synthesis and crystallization

The two compounds were obtained as by-products in crystal growth experiments aimed at synthesizing silicates from the quaternary Na_2_O–K_2_O–CaO–SiO_2_ system. A total of 0.5 g of the nutrient, composed of Na_2_O:K_2_O:CaO:SiO_2_ in a molar ratio of 1:1:6:12, was thoroughly homogenized in an agate mortar with 2.5 g of a Na_2_MoO_4_–K_2_MoO_4_ fluxing agent (molar ratio 1:1). The sample was then heated in a covered platinum crucible from room temperature to 1373 K at a heating rate of 2 K min^−1^. Following a three-day holding period at the maximum temperature, the sample was cooled to 1023 K at a rate of 0.1 K min^−1^. The crucible was then removed and quenched in air to ambient conditions. Following mechanical removal of the melt cake, the silicate phases were separated by dissolving the flux in distilled water on a watch glass at 295 K and 43% relative humidity (RH). New crystals were formed spontaneously in the remaining solution, which had been saturated with alkali molybdates, through slow evaporation of the solvent over the course of several hours. The presence of two distinct birefringent phases was indicated by differences in morphology (laths, plates). This was subsequently confirmed by single-crystal diffraction experiments. Following exposure to air (295 K, 43% RH) for a period of several hours, the initially transparent lath-shaped crystals of phase 1 [Na_3_K(MoO_4_)_2_(H_2_O)_9_] exhibited a transition in colour to an opaque hue, suggesting a gradual deterioration due to an ongoing dehydration process.

## 5. Refinement

Crystal data, data collection and structure refinement details are summarized in Table 7 ▸. To prevent possible water release, data acquisitions were performed at 193 K. Unconstrained site-population refinements of the K/Na populations on the relevant sites under the assumption of full occupancy did not show any indications for cation substitutions between the alkali atoms and, therefore, the non-tetra­hedral cation positions were occupied with either Na or K, respectively. Difference-Fourier calculations were employed to reveal the positions of the hydrogen atoms. This procedure allowed the location of the hydrogen atoms of all water sites in the asymmetric units of both phases. The positional parameters of the H-atoms were further optimized by a riding model with water-mol­ecule geometries restrained by DFIX 0.86 0.01 commands for the O—H and DFIX 1.35 0.02 commands for the H⋯H distances (giving H—O—H angles close to 105°). The isotropic displacement parameters for the H atoms of the water mol­ecules were coupled to those of the corresponding oxygen atoms according to *U*_iso_(H) = 1.2×*U*_eq_(O). The Flack parameter of acentric NaK(MoO_4_)(H_2_O) indicates that the absolute structure has been determined correctly (Table 7 ▸).

## Supplementary Material

Crystal structure: contains datablock(s) global, Na3KMoO42H2O9, NaKMoO4H2O. DOI: 10.1107/S2056989026004779/wm5795sup1.cif

CCDC references: 2552250, 2552249

Additional supporting information:  crystallographic information; 3D view; checkCIF report

## Figures and Tables

**Table 1:** Selected geometric parameters (Å, °) for Na_3_K(MoO_4_)_2_(H_2_O)_9_

Mo—O1^i^	1.753 (3)	Na—O1	2.418 (2)
Mo—O2^ii^	1.7683 (16)	Na—O3*W*^v^	2.437 (3)
Mo—O2	1.7683 (16)	K—O4*W*^vi^	2.8381 (19)
Mo—O2^iii^	1.7683 (16)	K—O4*W*^iv^	2.8381 (19)
Na—O4*W*	2.343 (2)	K—O4*W*^vii^	2.8381 (19)
Na—O4*W*^iv^	2.343 (2)	K—O4*W*^viii^	2.8381 (19)
Na—O3*W*	2.392 (3)	K—O4*W*	2.8381 (19)
Na—O1^v^	2.418 (2)	K—O4*W*^ix^	2.8381 (19)
			
O1^i^—Mo—O2^ii^	109.13 (6)	O3*W*—Na—O3*W*^v^	157.63 (10)
O1^i^—Mo—O2	109.13 (6)	O1^v^—Na—O3*W*^v^	81.06 (6)
O2^ii^—Mo—O2	109.81 (6)	O1—Na—O3*W*^v^	81.06 (6)
O1^i^—Mo—O2^iii^	109.13 (6)	O4*W*^vi^—K—O4*W*^iv^	131.88 (2)
O2^ii^—Mo—O2^iii^	109.81 (6)	O4*W*^vi^—K—O4*W*^vii^	70.73 (8)
O2—Mo—O2^iii^	109.81 (6)	O4*W*^iv^—K—O4*W*^vii^	89.86 (6)
O4*W*—Na—O4*W*^iv^	89.02 (10)	O4*W*^vi^—K—O4*W*^viii^	131.88 (2)
O4*W*—Na—O3*W*	94.79 (8)	O4*W*^iv^—K—O4*W*^viii^	89.86 (6)
O4*W*^iv^—Na—O3*W*	94.79 (8)	O4*W*^vii^—K—O4*W*^viii^	89.86 (6)
O4*W*—Na—O1^v^	175.15 (8)	O4*W*^vi^—K—O4*W*	89.86 (5)
O4*W*^iv^—Na—O1^v^	94.84 (7)	O4*W*^iv^—K—O4*W*	70.73 (8)
O3*W*—Na—O1^v^	81.99 (6)	O4*W*^vii^—K—O4*W*	131.88 (2)
O4*W*—Na—O1	94.84 (7)	O4*W*^viii^—K—O4*W*	131.88 (2)
O4*W*^iv^—Na—O1	175.15 (8)	O4*W*^vi^—K—O4*W*^ix^	89.85 (5)
O3*W*—Na—O1	81.99 (6)	O4*W*^iv^—K—O4*W*^ix^	131.88 (2)
O1^v^—Na—O1	81.15 (11)	O4*W*^vii^—K—O4*W*^ix^	131.88 (2)
O4*W*—Na—O3*W*^v^	101.09 (8)	O4*W*^viii^—K—O4*W*^ix^	70.73 (8)
O4*W*^iv^—Na—O3*W*^v^	101.09 (8)	O4*W*—K—O4*W*^ix^	89.86 (6)

Symmetry codes: (i) 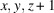; (ii) 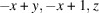; (iii) 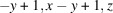; (iv) 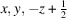; (v) 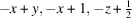; (vi) 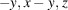; (vii) 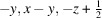; (viii) 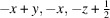; (ix) 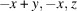.

**Table 2:** Hydrogen-bond geometry (Å, °) for Na_3_K(MoO_4_)_2_(H_2_O)_9_

*D*—H⋯*A*	*D*—H	H⋯*A*	*D*⋯*A*	*D*—H⋯*A*
O3*W*—H3⋯O2^x^	0.85 (1)	1.94 (1)	2.793 (2)	174 (3)
O4*W*—H41⋯O2^xi^	0.86 (1)	1.90 (1)	2.746 (2)	169 (3)
O4*W*—H42⋯O2^xii^	0.86 (1)	2.07 (1)	2.913 (3)	168 (3)

Symmetry codes: (x) 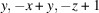; (xi) 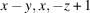; (xii) 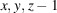.

**Table 3:** Selected geometric parameters (Å, °) for NaK(MoO_4_)(H_2_O)

Mo—O1	1.754 (3)	Na—O3^iii^	2.453 (3)
Mo—O2	1.755 (3)	K—O1^vii^	2.724 (3)
Mo—O4^i^	1.767 (2)	K—O2	2.791 (3)
Mo—O3^ii^	1.785 (3)	K—O3	2.798 (3)
Na—O5*W*^iii^	2.342 (3)	K—O3^viii^	2.840 (3)
Na—O4	2.352 (3)	K—O5*W*	2.919 (3)
Na—O1^iv^	2.406 (3)	K—O4^i^	2.930 (3)
Na—O5*W*^v^	2.411 (3)	K—O2^ii^	2.978 (3)
Na—O4^vi^	2.442 (3)	K—O1^ii^	3.198 (3)
			
O1—Mo—O2	107.95 (14)	O2—K—O3^viii^	131.11 (8)
O1—Mo—O4^i^	112.21 (13)	O3—K—O3^viii^	139.67 (4)
O2—Mo—O4^i^	105.53 (12)	O1^vii^—K—O5*W*	75.74 (8)
O1—Mo—O3^ii^	113.38 (12)	O2—K—O5*W*	168.15 (8)
O2—Mo—O3^ii^	110.03 (14)	O3—K—O5*W*	79.82 (8)
O4^i^—Mo—O3^ii^	107.46 (14)	O3^viii^—K—O5*W*	59.86 (7)
O5*W*^iii^—Na—O4	91.84 (12)	O1^vii^—K—O4^i^	71.77 (8)
O5*W*^iii^—Na—O1^iv^	81.07 (10)	O2—K—O4^i^	58.63 (8)
O4—Na—O1^iv^	88.45 (11)	O3—K—O4^i^	133.12 (9)
O5*W*^iii^—Na—O5*W*^v^	170.65 (10)	O3^viii^—K—O4^i^	81.38 (8)
O4—Na—O5*W*^v^	93.93 (11)	O5*W*—K—O4^i^	132.68 (7)
O1^iv^—Na—O5*W*^v^	91.72 (10)	O1^vii^—K—O2^ii^	146.17 (9)
O5*W*^iii^—Na—O4^vi^	93.35 (11)	O2—K—O2^ii^	81.77 (7)
O4—Na—O4^vi^	170.77 (10)	O3—K—O2^ii^	122.05 (8)
O1^iv^—Na—O4^vi^	84.82 (10)	O3^viii^—K—O2^ii^	69.32 (8)
O5*W*^v^—Na—O4^vi^	80.00 (11)	O5*W*—K—O2^ii^	100.67 (8)
O5*W*^iii^—Na—O3^iii^	99.81 (10)	O4^i^—K—O2^ii^	88.47 (8)
O4—Na—O3^iii^	86.15 (10)	O1^vii^—K—O1^ii^	142.92 (4)
O1^iv^—Na—O3^iii^	174.55 (13)	O2—K—O1^ii^	104.43 (8)
O5*W*^v^—Na—O3^iii^	87.93 (10)	O3—K—O1^ii^	73.36 (8)
O4^vi^—Na—O3^iii^	100.47 (11)	O3^viii^—K—O1^ii^	90.15 (9)
O1^vii^—K—O2	108.71 (9)	O5*W*—K—O1^ii^	68.64 (8)
O1^vii^—K—O3	90.85 (10)	O4^i^—K—O1^ii^	142.43 (8)
O2—K—O3	89.04 (8)	O2^ii^—K—O1^ii^	54.56 (7)
O1^vii^—K—O3^viii^	80.51 (9)		

Symmetry codes: (i) 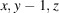; (ii) 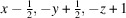; (iii) 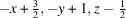; (iv) 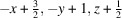; (v) 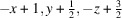; (vi) 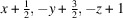; (vii) 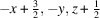; (viii) 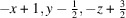.

**Table 4:** Hydrogen-bond geometry (Å, °) for NaK(MoO_4_)(H_2_O)

*D*—H⋯*A*	*D*—H	H⋯*A*	*D*⋯*A*	*D*—H⋯*A*
O5*W*—H51⋯O2^viii^	0.85 (1)	1.93 (2)	2.700 (4)	151 (4)
O5*W*—H52⋯O3^viii^	0.85 (1)	2.05 (2)	2.874 (4)	163 (4)

Symmetry code: (viii) 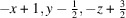.

**Table 5:** Bond-valence sums for Na_3_K(MoO_4_)_2_(H_2_O)_9_ in valence units (v.u.) The values in parentheses for the oxygen atoms refer to the calculations when O—H and O⋯H contributions are taken into consideration (see text).

	**Mo**	**Na**	**K**	**Sum**
**O1**	1.516	0.190^×2↓×3→^		2.087
**O2**	1.456^×3↓×1→^			1.456 (2.056)
**O3**		0.203; 0.180		0.383 (1.983)
**O4**		0.232^×2↓×1→^	0.148^×6↓×1→^	0.380 (1.981)
**Sum**	5.884	1.227	0.890	

**Table 6:** Bond valence sums for NaK(MoO_4_)(H_2_O) in valence units (v.u.) The values in parentheses for the oxygen atoms refer to the calculations when O—H and O⋯H contributions are taken into consideration (see text).

	**Mo**	**Na**	**K**	**Sum**
**O1**	1.512	0.196	0.202; 0.056	1.966
**O2**	1.508		0.168; 0.102	1.778 (1.978)
**O3**	1.391	0.173	0.165; 0.148	1.877 (2.077)
**O4**	1.450	0.227; 0.178	0.116	1.980
**O5**		0.233; 0.193	0.119	0.545 (2.146)
**Sum**	5.871	1.200	1.076	

**Table 7:** Experimental details

	Na_3_K(MoO_4_)_2_(H_2_O)_9_	NaK(MoO_4_)(H_2_O)
Crystal data		
*M*_r_	590.09	240.04
Crystal system, space group	Hexagonal, *P*6_3_/*m*	Orthorhombic, *P*2_1_2_1_2_1_
Temperature (K)	193	193
*a*, *b*, *c* (Å)	9.4974 (11), 9.4974 (11), 12.2139 (14)	6.4781 (8), 8.0697 (10), 10.1399 (13)
α, β, γ (°)	90, 90, 120	90, 90, 90
*V* (Å^3^)	954.10 (19)	530.08 (11)
*Z*	2	4
Radiation type	Mo *K*α	Mo *K*α
μ (mm^−1^)	1.67	3.27
Crystal size (mm)	0.43 × 0.09 × 0.06	0.18 × 0.14 × 0.06

Data collection		
Diffractometer	Xcalibur, Ruby, Gemini ultra	Xcalibur, Ruby, Gemini ultra
Absorption correction	Analytical [*CrysAlis PRO* (Rigaku OD, 2020 ▸). Analytical numeric absorption correction using a multifaceted crystal model based on expressions derived by (Clark & Reid, 1995 ▸)]	Analytical [*CrysAlis PRO* (Rigaku OD, 2020 ▸). Analytical numeric absorption correction using a multifaceted crystal model based on expressions derived by (Clark & Reid, 1995 ▸)]
*T*_min_, *T*_max_	0.981, 0.995	0.738, 0.869
No. of measured, independent and observed [*I* > 2σ(*I*)] reflections	7218, 800, 697	3887, 1249, 1208
*R*_int_	0.046	0.039
(sin θ/λ)_max_ (Å^−1^)	0.658	0.658

Refinement		
*R*[*F*^2^ > 2σ(*F*^2^)], *wR*(*F*^2^), *S*	0.022, 0.06, 1.09	0.024, 0.058, 1.07
No. of reflections	800	1249
No. of parameters	49	79
No. of restraints	5	3
H-atom treatment	H atoms treated by a mixture of independent and constrained refinement	H atoms treated by a mixture of independent and constrained refinement
Δρ_max_, Δρ_min_ (e Å^−3^)	1.16, −0.64	0.43, −0.87
Absolute structure	–	Flack (1983 ▸)
Absolute structure parameter	–	−0.01 (7)

Computer programs: *CrysAlis PRO* (Rigaku OD, 2020 ▸), *SIR2004* (Burla *et al.*, 2005 ▸), *SHELXL97* (Sheldrick, 2008 ▸), *VESTA-3* (Momma & Izumi, 2011 ▸) and *WinGX* (Farrugia, 2012 ▸).

## supplementary crystallographic information

### Trisodium potassium bis(orthomolybdate) nonahydrate (Na3KMoO42H2O9) Crystal data

**Table:**

Na_3_K(MoO_4_)_2_(H_2_O)_9_	*D*_x_ = 2.054 Mg m^−^^3^
*M**_r_* = 590.09	Mo *K*α radiation, λ = 0.71073 Å
Hexagonal, *P*6_3_/*m*	Cell parameters from 2517 reflections
Hall symbol: -P 6c	θ = 4.9–29.7°
*a* = 9.4974 (11) Å	µ = 1.67 mm^−^^1^
*c* = 12.2139 (14) Å	*T* = 193 K
*V* = 954.10 (19) Å^3^	Lath-shaped fragment, colourless
*Z* = 2	0.43 × 0.09 × 0.06 mm
*F*(000) = 580	

### Trisodium potassium bis(orthomolybdate) nonahydrate (Na3KMoO42H2O9) Data collection

**Table:**

Xcalibur, Ruby, Gemini ultra diffractometer	800 independent reflections
Radiation source: fine-focus sealed X-ray tube, Enhance (Mo) X-ray Source	697 reflections with *I* > 2σ(*I*)
Graphite monochromator	*R*_int_ = 0.046
Detector resolution: 10.3575 pixels mm^-1^	θ_max_ = 27.9°, θ_min_ = 3.3°
ω scans	*h* = −12→12
Absorption correction: analytical [CrysAlisPro (Rigaku OD, 2020). Analytical numeric absorption correction using a multifaceted crystal model based on expressions derived by (Clark & Reid, 1995)]	*k* = −11→12
*T*_min_ = 0.981, *T*_max_ = 0.995	*l* = −16→15
7218 measured reflections	

### Trisodium potassium bis(orthomolybdate) nonahydrate (Na3KMoO42H2O9) Refinement

**Table:**

Refinement on *F*^2^	Secondary atom site location: difference Fourier map
Least-squares matrix: full	Hydrogen site location: mixed
*R*[*F*^2^ > 2σ(*F*^2^)] = 0.022	H atoms treated by a mixture of independent and constrained refinement
*wR*(*F*^2^) = 0.06	*w* = 1/[σ^2^(*F*_o_^2^) + (0.0297*P*)^2^ + 0.6304*P*] where *P* = (*F*_o_^2^ + 2*F*_c_^2^)/3
*S* = 1.09	(Δ/σ)_max_ = 0.001
800 reflections	Δρ_max_ = 1.16 e Å^−^^3^
49 parameters	Δρ_min_ = −0.64 e Å^−^^3^
5 restraints	Extinction correction: SHELXL, Fc^*^=kFc[1+0.001xFc^2^λ^3^/sin(2θ)]^-1/4^
Primary atom site location: structure-invariant direct methods	Extinction coefficient: 0.0051 (10)

### Trisodium potassium bis(orthomolybdate) nonahydrate (Na3KMoO42H2O9) Special details

**Table:**

Geometry. All s.u.'s (except the s.u. in the dihedral angle between two l.s. planes) are estimated using the full covariance matrix. The cell s.u.'s are taken into account individually in the estimation of s.u.'s in distances, angles and torsion angles; correlations between s.u.'s in cell parameters are only used when they are defined by crystal symmetry. An approximate (isotropic) treatment of cell s.u.'s is used for estimating s.u.'s involving l.s. planes.
Refinement. Refinement of F^2^ against ALL reflections. The weighted R-factor wR and goodness of fit S are based on F^2^, conventional R-factors R are based on F, with F set to zero for negative F^2^. The threshold expression of F^2^ > 2σ(F^2^) is used only for calculating R-factors(gt) etc. and is not relevant to the choice of reflections for refinement. R-factors based on F^2^ are statistically about twice as large as those based on F, and R- factors based on ALL data will be even larger.

### Trisodium potassium bis(orthomolybdate) nonahydrate (Na3KMoO42H2O9) Fractional atomic coordinates and isotropic or equivalent isotropic displacement parameters (Å^2^)

**Table:**

	*x*	*y*	*z*	*U*_iso_*/*U*_eq_	
Mo	0.33333	0.66667	0.97769 (2)	0.01574 (14)	
Na	0.16850 (16)	0.45384 (16)	0.25	0.0222 (3)	
K	0	0	0.25	0.0362 (3)	
O1	0.33333	0.66667	0.1213 (2)	0.0190 (6)	
O2	0.1881 (2)	0.4711 (2)	0.93025 (14)	0.0260 (4)	
O3W	0.4008 (3)	0.4183 (3)	0.25	0.0275 (5)	
H3	0.428 (3)	0.383 (3)	0.1942 (4)	0.033*	
O4W	0.0281 (2)	0.2565 (2)	0.11551 (15)	0.0320 (4)	
H41	−0.0704 (15)	0.236 (4)	0.110 (2)	0.038*	
H42	0.074 (3)	0.307 (4)	0.0563 (16)	0.038*	

### Trisodium potassium bis(orthomolybdate) nonahydrate (Na3KMoO42H2O9) Atomic displacement parameters (Å^2^)

**Table:**

	*U*^11^	*U*^22^	*U*^33^	*U*^12^	*U*^13^	*U*^23^
Mo	0.01825 (16)	0.01825 (16)	0.0107 (2)	0.00912 (8)	0	0
Na	0.0222 (7)	0.0200 (6)	0.0219 (6)	0.0086 (5)	0	0
K	0.0325 (5)	0.0325 (5)	0.0438 (8)	0.0162 (2)	0	0
O1	0.0210 (9)	0.0210 (9)	0.0152 (13)	0.0105 (4)	0	0
O2	0.0277 (9)	0.0240 (9)	0.0233 (9)	0.0106 (7)	−0.0061 (7)	−0.0050 (7)
O3W	0.0396 (15)	0.0378 (14)	0.0172 (11)	0.0283 (13)	0	0
O4W	0.0249 (9)	0.0384 (11)	0.0281 (9)	0.0125 (9)	−0.0010 (8)	−0.0002 (8)

### Trisodium potassium bis(orthomolybdate) nonahydrate (Na3KMoO42H2O9) Geometric parameters (Å, º)

**Table:**

Mo—O1^i^	1.753 (3)	Na—O1	2.418 (2)
Mo—O2^ii^	1.7683 (16)	Na—O3W^v^	2.437 (3)
Mo—O2	1.7683 (16)	K—O4W^vi^	2.8381 (19)
Mo—O2^iii^	1.7683 (16)	K—O4W^iv^	2.8381 (19)
Na—O4W	2.343 (2)	K—O4W^vii^	2.8381 (19)
Na—O4W^iv^	2.343 (2)	K—O4W^viii^	2.8381 (19)
Na—O3W	2.392 (3)	K—O4W	2.8381 (19)
Na—O1^v^	2.418 (2)	K—O4W^ix^	2.8381 (19)
			
O1^i^—Mo—O2^ii^	109.13 (6)	O3W—Na—O3W^v^	157.63 (10)
O1^i^—Mo—O2	109.13 (6)	O1^v^—Na—O3W^v^	81.06 (6)
O2^ii^—Mo—O2	109.81 (6)	O1—Na—O3W^v^	81.06 (6)
O1^i^—Mo—O2^iii^	109.13 (6)	O4W^vi^—K—O4W^iv^	131.88 (2)
O2^ii^—Mo—O2^iii^	109.81 (6)	O4W^vi^—K—O4W^vii^	70.73 (8)
O2—Mo—O2^iii^	109.81 (6)	O4W^iv^—K—O4W^vii^	89.86 (6)
O4W—Na—O4W^iv^	89.02 (10)	O4W^vi^—K—O4W^viii^	131.88 (2)
O4W—Na—O3W	94.79 (8)	O4W^iv^—K—O4W^viii^	89.86 (6)
O4W^iv^—Na—O3W	94.79 (8)	O4W^vii^—K—O4W^viii^	89.86 (6)
O4W—Na—O1^v^	175.15 (8)	O4W^vi^—K—O4W	89.86 (5)
O4W^iv^—Na—O1^v^	94.84 (7)	O4W^iv^—K—O4W	70.73 (8)
O3W—Na—O1^v^	81.99 (6)	O4W^vii^—K—O4W	131.88 (2)
O4W—Na—O1	94.84 (7)	O4W^viii^—K—O4W	131.88 (2)
O4W^iv^—Na—O1	175.15 (8)	O4W^vi^—K—O4W^ix^	89.85 (5)
O3W—Na—O1	81.99 (6)	O4W^iv^—K—O4W^ix^	131.88 (2)
O1^v^—Na—O1	81.15 (11)	O4W^vii^—K—O4W^ix^	131.88 (2)
O4W—Na—O3W^v^	101.09 (8)	O4W^viii^—K—O4W^ix^	70.73 (8)
O4W^iv^—Na—O3W^v^	101.09 (8)	O4W—K—O4W^ix^	89.86 (6)

Symmetry codes: (i) *x*, *y*, *z*+1; (ii) −*x*+*y*, −*x*+1, *z*; (iii) −*y*+1, *x*−*y*+1, *z*; (iv) *x*, *y*, −*z*+1/2; (v) −*x*+*y*, −*x*+1, −*z*+1/2; (vi) −*y*, *x*−*y*, *z*; (vii) −*y*, *x*−*y*, −*z*+1/2; (viii) −*x*+*y*, −*x*, −*z*+1/2; (ix) −*x*+*y*, −*x*, *z*.

### Trisodium potassium bis(orthomolybdate) nonahydrate (Na3KMoO42H2O9) Hydrogen-bond geometry (Å, º)

**Table:**

*D*—H···*A*	*D*—H	H···*A*	*D*···*A*	*D*—H···*A*
O3*W*—H3···O2^x^	0.85 (1)	1.94 (1)	2.793 (2)	174 (3)
O4*W*—H41···O2^xi^	0.86 (1)	1.90 (1)	2.746 (2)	169 (3)
O4*W*—H42···O2^xii^	0.86 (1)	2.07 (1)	2.913 (3)	168 (3)

Symmetry codes: (x) *y*, −*x*+*y*, −*z*+1; (xi) *x*−*y*, *x*, −*z*+1; (xii) *x*, *y*, *z*−1.

### Sodium potassium orthomolybdate monohydrate (NaKMoO4H2O) Crystal data

**Table:**

NaK(MoO_4_)(H_2_O)	*F*(000) = 456
*M**_r_* = 240.04	*D*_x_ = 3.008 Mg m^−^^3^
Orthorhombic, *P*2_1_2_1_2_1_	Mo *K*α radiation, λ = 0.71073 Å
Hall symbol: P 2ac 2ab	Cell parameters from 1970 reflections
*a* = 6.4781 (8) Å	θ = 5.6–29.5°
*b* = 8.0697 (10) Å	µ = 3.27 mm^−^^1^
*c* = 10.1399 (13) Å	*T* = 193 K
*V* = 530.08 (11) Å^3^	Irregular fragment, colourless
*Z* = 4	0.18 × 0.14 × 0.06 mm

### Sodium potassium orthomolybdate monohydrate (NaKMoO4H2O) Data collection

**Table:**

Xcalibur, Ruby, Gemini ultra diffractometer	1249 independent reflections
Radiation source: fine-focus sealed X-ray tube, Enhance (Mo) X-ray Source	1208 reflections with *I* > 2σ(*I*)
Graphite monochromator	*R*_int_ = 0.039
Detector resolution: 10.3575 pixels mm^-1^	θ_max_ = 27.9°, θ_min_ = 3.7°
ω scans	*h* = −8→8
Absorption correction: analytical [CrysAlisPro (Rigaku OD, 2020). Analytical numeric absorption correction using a multifaceted crystal model based on expressions derived by (Clark & Reid, 1995)]	*k* = −10→10
*T*_min_ = 0.738, *T*_max_ = 0.869	*l* = −13→13
3887 measured reflections	

### Sodium potassium orthomolybdate monohydrate (NaKMoO4H2O) Refinement

**Table:**

Refinement on *F*^2^	Secondary atom site location: difference Fourier map
Least-squares matrix: full	Hydrogen site location: mixed
*R*[*F*^2^ > 2σ(*F*^2^)] = 0.024	H atoms treated by a mixture of independent and constrained refinement
*wR*(*F*^2^) = 0.058	*w* = 1/[σ^2^(*F*_o_^2^) + (0.0269*P*)^2^] where *P* = (*F*_o_^2^ + 2*F*_c_^2^)/3
*S* = 1.07	(Δ/σ)_max_ = 0.001
1249 reflections	Δρ_max_ = 0.43 e Å^−^^3^
79 parameters	Δρ_min_ = −0.87 e Å^−^^3^
3 restraints	Absolute structure: Flack (1983)
Primary atom site location: structure-invariant direct methods	Absolute structure parameter: −0.01 (7)

### Sodium potassium orthomolybdate monohydrate (NaKMoO4H2O) Special details

**Table:**

Geometry. All s.u.'s (except the s.u. in the dihedral angle between two l.s. planes) are estimated using the full covariance matrix. The cell s.u.'s are taken into account individually in the estimation of s.u.'s in distances, angles and torsion angles; correlations between s.u.'s in cell parameters are only used when they are defined by crystal symmetry. An approximate (isotropic) treatment of cell s.u.'s is used for estimating s.u.'s involving l.s. planes.
Refinement. Refinement of F^2^ against ALL reflections. The weighted R-factor wR and goodness of fit S are based on F^2^, conventional R-factors R are based on F, with F set to zero for negative F^2^. The threshold expression of F^2^ > 2σ(F^2^) is used only for calculating R-factors(gt) etc. and is not relevant to the choice of reflections for refinement. R-factors based on F^2^ are statistically about twice as large as those based on F, and R- factors based on ALL data will be even larger.

### Sodium potassium orthomolybdate monohydrate (NaKMoO4H2O) Fractional atomic coordinates and isotropic or equivalent isotropic displacement parameters (Å^2^)

**Table:**

	*x*	*y*	*z*	*U*_iso_*/*U*_eq_	
Mo	0.51460 (5)	0.11176 (3)	0.30697 (3)	0.00784 (10)	
Na	0.7761 (2)	0.7544 (2)	0.48190 (14)	0.0127 (3)	
K	0.49819 (15)	0.13919 (9)	0.65476 (8)	0.01524 (18)	
O1	0.7135 (4)	0.1024 (4)	0.1899 (2)	0.0156 (6)	
O2	0.5594 (5)	0.2846 (3)	0.4081 (2)	0.0181 (7)	
O3	0.7634 (5)	0.3755 (4)	0.7646 (2)	0.0136 (6)	
O4	0.5160 (5)	0.9389 (3)	0.4140 (2)	0.0140 (5)	
O5W	0.4645 (5)	0.0528 (3)	0.9335 (2)	0.0131 (6)	
H51	0.500 (6)	−0.022 (3)	0.987 (3)	0.016*	
H52	0.376 (5)	0.009 (4)	0.883 (3)	0.016*	

### Sodium potassium orthomolybdate monohydrate (NaKMoO4H2O) Atomic displacement parameters (Å^2^)

**Table:**

	*U*^11^	*U*^22^	*U*^33^	*U*^12^	*U*^13^	*U*^23^
Mo	0.00733 (15)	0.00843 (15)	0.00776 (16)	−0.00002 (14)	0.00045 (13)	0.00036 (10)
Na	0.0095 (8)	0.0153 (7)	0.0133 (7)	−0.0015 (6)	−0.0009 (6)	−0.0002 (7)
K	0.0175 (4)	0.0158 (3)	0.0124 (4)	−0.0007 (4)	−0.0005 (4)	0.0013 (3)
O1	0.0144 (13)	0.0201 (15)	0.0122 (12)	0.0029 (13)	0.0035 (11)	0.0066 (15)
O2	0.0266 (19)	0.0115 (12)	0.0163 (13)	−0.0028 (12)	−0.0006 (13)	−0.0030 (11)
O3	0.0114 (14)	0.0158 (15)	0.0137 (13)	−0.0025 (12)	0.0026 (10)	0.0014 (13)
O4	0.0150 (15)	0.0122 (11)	0.0149 (11)	−0.0003 (13)	0.0007 (13)	0.0056 (10)
O5W	0.0136 (15)	0.0134 (12)	0.0121 (12)	−0.0022 (12)	−0.0044 (11)	0.0016 (10)

### Sodium potassium orthomolybdate monohydrate (NaKMoO4H2O) Geometric parameters (Å, º)

**Table:**

Mo—O1	1.754 (3)	Na—O3^iii^	2.453 (3)
Mo—O2	1.755 (3)	K—O1^vii^	2.724 (3)
Mo—O4^i^	1.767 (2)	K—O2	2.791 (3)
Mo—O3^ii^	1.785 (3)	K—O3	2.798 (3)
Na—O5W^iii^	2.342 (3)	K—O3^viii^	2.840 (3)
Na—O4	2.352 (3)	K—O5W	2.919 (3)
Na—O1^iv^	2.406 (3)	K—O4^i^	2.930 (3)
Na—O5W^v^	2.411 (3)	K—O2^ii^	2.978 (3)
Na—O4^vi^	2.442 (3)	K—O1^ii^	3.198 (3)
			
O1—Mo—O2	107.95 (14)	O2—K—O3^viii^	131.11 (8)
O1—Mo—O4^i^	112.21 (13)	O3—K—O3^viii^	139.67 (4)
O2—Mo—O4^i^	105.53 (12)	O1^vii^—K—O5W	75.74 (8)
O1—Mo—O3^ii^	113.38 (12)	O2—K—O5W	168.15 (8)
O2—Mo—O3^ii^	110.03 (14)	O3—K—O5W	79.82 (8)
O4^i^—Mo—O3^ii^	107.46 (14)	O3^viii^—K—O5W	59.86 (7)
O5W^iii^—Na—O4	91.84 (12)	O1^vii^—K—O4^i^	71.77 (8)
O5W^iii^—Na—O1^iv^	81.07 (10)	O2—K—O4^i^	58.63 (8)
O4—Na—O1^iv^	88.45 (11)	O3—K—O4^i^	133.12 (9)
O5W^iii^—Na—O5W^v^	170.65 (10)	O3^viii^—K—O4^i^	81.38 (8)
O4—Na—O5W^v^	93.93 (11)	O5W—K—O4^i^	132.68 (7)
O1^iv^—Na—O5W^v^	91.72 (10)	O1^vii^—K—O2^ii^	146.17 (9)
O5W^iii^—Na—O4^vi^	93.35 (11)	O2—K—O2^ii^	81.77 (7)
O4—Na—O4^vi^	170.77 (10)	O3—K—O2^ii^	122.05 (8)
O1^iv^—Na—O4^vi^	84.82 (10)	O3^viii^—K—O2^ii^	69.32 (8)
O5W^v^—Na—O4^vi^	80.00 (11)	O5W—K—O2^ii^	100.67 (8)
O5W^iii^—Na—O3^iii^	99.81 (10)	O4^i^—K—O2^ii^	88.47 (8)
O4—Na—O3^iii^	86.15 (10)	O1^vii^—K—O1^ii^	142.92 (4)
O1^iv^—Na—O3^iii^	174.55 (13)	O2—K—O1^ii^	104.43 (8)
O5W^v^—Na—O3^iii^	87.93 (10)	O3—K—O1^ii^	73.36 (8)
O4^vi^—Na—O3^iii^	100.47 (11)	O3^viii^—K—O1^ii^	90.15 (9)
O1^vii^—K—O2	108.71 (9)	O5W—K—O1^ii^	68.64 (8)
O1^vii^—K—O3	90.85 (10)	O4^i^—K—O1^ii^	142.43 (8)
O2—K—O3	89.04 (8)	O2^ii^—K—O1^ii^	54.56 (7)
O1^vii^—K—O3^viii^	80.51 (9)		

Symmetry codes: (i) *x*, *y*−1, *z*; (ii) *x*−1/2, −*y*+1/2, −*z*+1; (iii) −*x*+3/2, −*y*+1, *z*−1/2; (iv) −*x*+3/2, −*y*+1, *z*+1/2; (v) −*x*+1, *y*+1/2, −*z*+3/2; (vi) *x*+1/2, −*y*+3/2, −*z*+1; (vii) −*x*+3/2, −*y*, *z*+1/2; (viii) −*x*+1, *y*−1/2, −*z*+3/2.

### Sodium potassium orthomolybdate monohydrate (NaKMoO4H2O) Hydrogen-bond geometry (Å, º)

**Table:**

*D*—H···*A*	*D*—H	H···*A*	*D*···*A*	*D*—H···*A*
O5*W*—H51···O2^viii^	0.85 (1)	1.93 (2)	2.700 (4)	151 (4)
O5*W*—H52···O3^viii^	0.85 (1)	2.05 (2)	2.874 (4)	163 (4)

Symmetry code: (viii) −*x*+1, *y*−1/2, −*z*+3/2.
